# Contribution of plasma cells and B cells to hidradenitis suppurativa pathogenesis

**DOI:** 10.1172/jci.insight.139930

**Published:** 2020-10-02

**Authors:** Johann E. Gudjonsson, Lam C. Tsoi, Feiyang Ma, Allison C. Billi, K.R. van Straalen, A.R.J.V. Vossen, H.H. van der Zee, Paul W. Harms, Rachael Wasikowski, Christine M. Yee, Syed M. Rizvi, Xianying Xing, Enze Xing, Olesya Plazyo, Chang Zeng, Matthew T. Patrick, Margaret M. Lowe, Richard E. Burney, Jeffrey H. Kozlow, Jill R. Cherry-Bukowiec, Yanyun Jiang, Joseph Kirma, Stephan Weidinger, Kelly C. Cushing, Michael D. Rosenblum, Celine Berthier, Amanda S. MacLeod, John J. Voorhees, Fei Wen, J. Michelle Kahlenberg, Emanual Maverakis, Robert L. Modlin, Errol P. Prens

**Affiliations:** 1 Department of Dermatology and; 2 Department of Computational Medicine and Bioinformatics, University of Michigan Medical School, Ann Arbor, Michigan, USA.; 3 Department of Biostatistics, School of Public Health, University of Michigan, Ann Arbor, Michigan, USA.; 4 Department of Molecular, Cell and Developmental Biology, David Geffen School of Medicine at University of California (UCLA), Los Angeles, California, USA.; 5 Department of Dermatology, Erasmus University Medical Center, Rotterdam, Netherlands.; 6 Department of Pathology, University of Michigan Medical School, Ann Arbor, Michigan, USA.; 7 Department of Chemical Engineering, University of Michigan, Ann Arbor, Michigan, USA.; 8 Department of Dermatology, UCSF, San Francisco, California, USA.; 9 Department of Surgery,; 10 Section of Plastic Surgery, Department of Surgery, and; 11 Department of Surgery, University of Michigan Medical School, Ann Arbor, Michigan, USA.; 12 Department of Dermatology, Venereology and Allergy, University Hospital Schleswig-Holstein, Campus Kiel, Kiel, Germany.; 13 Division of Gastroenterology and Hepatology and; 14 Division of Nephrology, Department of Internal Medicine, University of Michigan Medical School, Ann Arbor, Michigan, USA.; 15 Department of Dermatology, Duke University School of Medicine, Durham, North Carolina, USA.; 16 Division of Rheumatology, Department of Internal Medicine, University of Michigan, Ann Arbor, Michigan, USA.; 17 Department of Dermatology, University of California Davis School of Medicine, Sacramento, California, USA.; 18 Division of Dermatology, Department of Medicine, Immunology and Molecular Genetics, David Geffen School of Medicine at UCLA, Los Angeles, California, USA.

**Keywords:** Dermatology, Immunology, B cells, Complement, Skin

## Abstract

Hidradenitis suppurativa (HS) is a debilitating chronic inflammatory skin disease characterized by chronic abscess formation and development of multiple draining sinus tracts in the groin, axillae, and perineum. Using proteomic and transcriptomic approaches, we characterized the inflammatory responses in HS in depth, revealing immune responses centered on IFN-γ, IL-36, and TNF, with lesser contribution from IL-17A. We further identified B cells and plasma cells, with associated increases in immunoglobulin production and complement activation, as pivotal players in HS pathogenesis, with Bruton’s tyrosine kinase (BTK) and spleen tyrosine kinase (SYK) pathway activation as a central signal transduction network in HS. These data provide preclinical evidence to accelerate the path toward clinical trials targeting BTK and SYK signaling in moderate-to-severe HS.

## Abstract 2

B-cells and plasma cells are critical pathogenic cell populations in chronic Hidradenitis Suppurativa and are potential therapeutic targets.

## Introduction

Hidradenitis suppurativa (HS) is a debilitating chronic inflammatory skin disease characterized by chronic abscess formation and development of multiple draining sinus tracts in the groin, axillae, and perineum (1). It has an estimated prevalence of approximately 1% (2) and is more common in women and specific ethnic groups, such as African Americans (3), in which the incidence may be up to 3-fold higher than in Caucasians (4). It frequently arises during adolescence and is a lifelong disease with a marked negative effect on quality of life (1). It is associated with several comorbidities, including obesity, and has a prominent hormonal component (5). Several genetic mutations involving 3 gamma-secretase complex genes have been identified in autosomal dominant forms of HS (6), forming the basis for the hypothesis that Notch signaling is involved in HS pathogenesis (7). However, these mutations account for only a small minority of HS cases (7).

The immunopathogenesis of HS is poorly understood, with widely and somewhat discordant pathogenic mechanisms proposed. Complement activation has been implicated in the pathogenesis of HS through elevated concentration of the complement fragment C5a in skin and blood of patients with HS (8). C5a is a major chemoattractant for neutrophils, a cell population that is prominent in actively inflamed HS skin (9). Furthermore, increased levels of neutrophils releasing neutrophil extracellular traps (NETs) in a process called NETosis have been demonstrated in both blood and skin of patients with HS (10), along with increased type I IFN responses and activation of plasmacytoid dendritic cells (pDCs) (10).

Other studies have suggested involvement of the proinflammatory cytokines IL-17A and IFN-γ (11, 12), as well as keratinocyte-derived IL-6 and IL-1β (11). Additional transcriptomic studies have noted the increased expression of immunoglobulins and plasma cell signatures in HS (13), but a clear and comprehensive view of the immunopathogenic mechanisms involved in HS has been lacking. HS remains difficult to treat, with only one biologic therapy, the anti-TNF agent adalimumab, currently approved for its treatment, and even with adalimumab therapeutic responses are suboptimal in nearly 40% of patients (14). Therefore, there is an urgent need for increasing our understanding of this disease to facilitate therapeutic development.

In this manuscript we provide potentially novel insights into the immunopathogenesis of HS, using bulk and single-cell RNA-sequencing and cytometry by time of flight (CyTOF) imaging to outline the major cell types and inflammatory pathways that are dysregulated in HS skin. Our results identify immune complex deposition and complement activation along with B cell and plasma cell activation as critical pathways in HS pathogenesis and provide data suggesting that targeting plasma cell activation could provide a novel therapeutic approach for treating advanced HS.

## Results

### HS is characterized by enriched biological processes involving complement and B cell responses.

**Figure 1:**
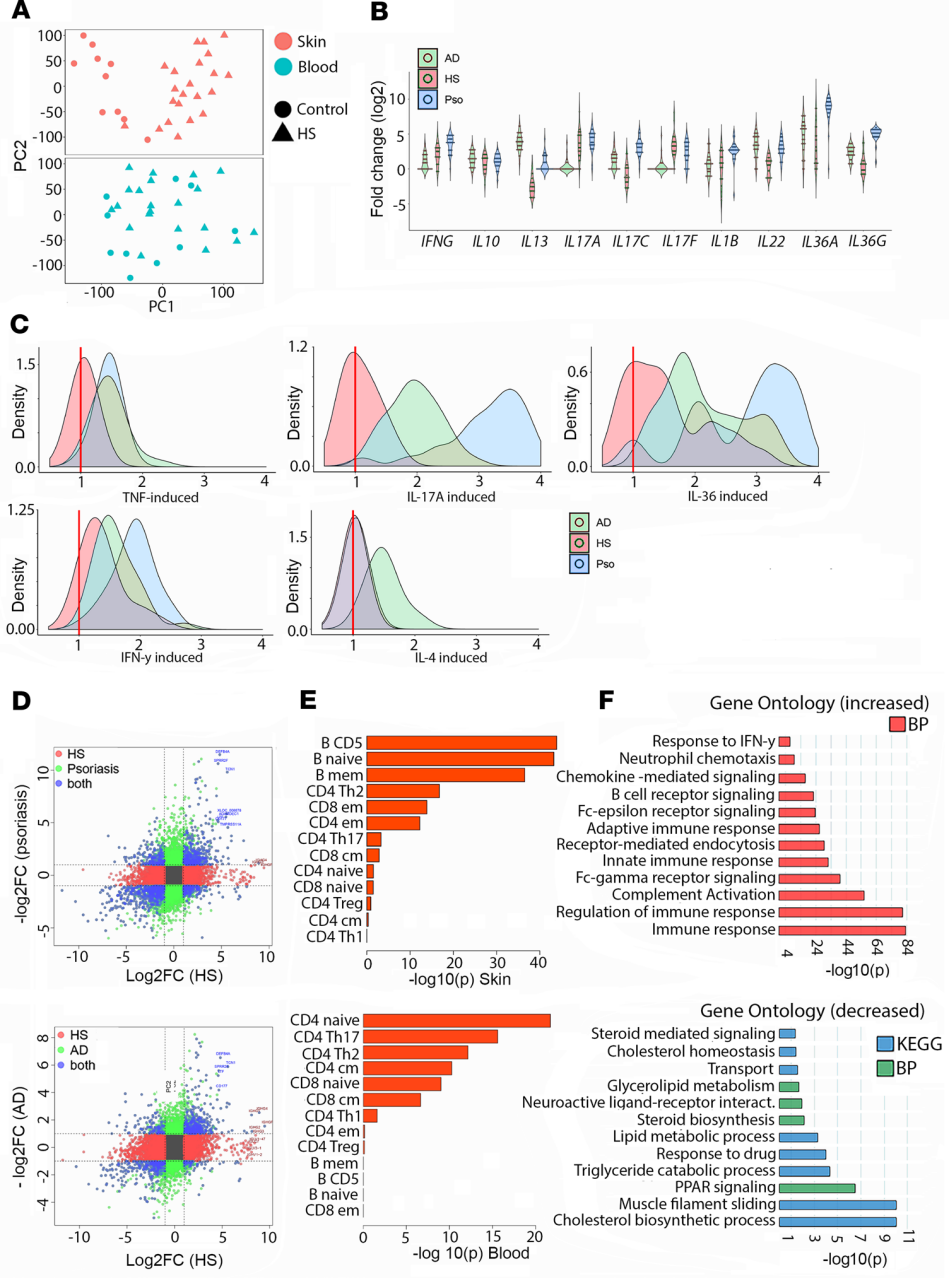
Characterization of the inflammatory process in HS by RNA-Seq is suggestive of heightened B cell responses. PCA plots of skin (top, red), and blood (bottom, blue) in patients with HS (*n* = 22) and healthy controls (*n* = 10) (**A**). Comparison of fold change mRNA expression of key proinflammatory cytokines in HS compared with psoriasis and AD (*n* = 22 HS, *n* = 28 psoriasis, *n* = 32 AD). Medians are shown in the middle of each plot. (**B**). Comparison of key proinflammatory cytokine responses in HS skin compared with psoriasis and AD. (*n* = 22 HS, *n* = 28 psoriasis, *n* = 32 AD) (red bar indicates baseline responses in uninflamed control skin) (**C**). Comparison of DEGs in HS skin against psoriasis (*n* = 28) and AD (*n* = 32). Unique genes in HS are shown in red, genes unique to psoriasis or AD are shown in green, and genes significant in both are shown in blue (**D**). Enriched B cell signatures in skin of patients with HS but T cell responses in blood of patients with HS (**E**). Enriched biological processes and Kyoto Encyclopedia of Genes and Genomes (KEGG) pathways in increased (top) and decreased (bottom) DEGs in HS skin (**F**).

Twenty-two patients with moderate-to-severe HS and 10 healthy controls (see Supplemental Table 1; supplemental material available online with this article; https://doi.org/10.1172/jci.insight.139930DS1) were enrolled, and lesional (HS) and matching control skin samples were obtained and submitted for bulk RNA-Seq. In addition, RNA-Seq of whole blood (WB) cells was performed on 21 HS patients and 10 healthy controls, with 20 of the HS patients and all the healthy controls having both skin and WB samples profiled. Principal components analyses (PCAs) of skin showed nearly complete separation between HS and healthy control skin (Figure 1A), whereas PBMCs showed less clear separation (Figure 1A). Using a fold change threshold of 2 and false discovery rate (FDR) of 0.1, we detected 4797 differentially expressed genes (DEGs) in HS skin compared with control skin, of which 2584 were increased and 2213 were decreased. Several genes were found to be increased simultaneously in both HS skin and HS blood. These included *CCR4*, *TNFRSF4*, and several genes involved in immunoglobulin biosynthesis (Supplemental Figure 1). The most upregulated genes in skin were associated with B cell responses, including immunoglobulin genes such as *IGLV3-27* (100-fold increased, adjusted *P* = 2.74 × 10^–5^), *CD19* (33-fold, adjusted *P* = 6.48 × 10^–24^), and *CD79a* (32-fold, adjusted *P* = 3.58 × 10^–22^). Other genes included the antimicrobial gene *DEFB4A* (24-fold, adjusted *P* = 2.71 × 10^–10^); *CXCL13*, a B cell chemoattractant (16-fold, adjusted *P* = 1.25 × 10^–8^); and the neutrophil chemokine *CXCL1* (2.8-fold, adjusted *P* = 2.91 × 10^–2^). In the WB, there were 332 DEGs, of which 230 were increased and 102 decreased (Supplemental Tables 2 and 3).

### HS shows a complex inflammatory profile distinct from that of psoriasis or atopic dermatitis and enriched in genes involved in B cell function.

To address the major transcriptomic characteristics of HS, we compared it with RNA-Seq data from psoriasis (*n* = 28) and atopic dermatitis (AD) (*n* = 32) (15) because the inflammatory responses in these 2 diseases are well characterized, and many of the drugs currently approved for these diseases are currently being repurposed for treatment of HS. Interestingly, genes dysregulated in lesional skin for all 3 diseases included the antimicrobial genes *DEFB4A*, *SPRR2F*, *CD177*, and *TCN1*. In terms of expression of proinflammatory cytokine genes in HS, we observed increased mRNA expression of *IFNG* (2.6-fold, adjusted *P* = 2.6 × 10^–2^), *IL17A* (8.6-fold, adjusted *P* = 6.7 × 10^–7^), *IL17F* (13.3-fold, adjusted *P* = 1.9 × 10^–9^), *IL36A* (9-fold, adjusted *P* = 1.2 × 10^–4^), and *IL36G* (2.4-fold, adjusted *P* = 1.7 × 10^–2^) compared with healthy controls, whereas *IL13* and *IL17C* expression were overall decreased (Figure 1B and Supplemental Figure 2). Notably, the elevation of *IL17A* and *IL17F* expression in HS was comparable to the expression levels in psoriatic skin. With regard to the magnitude of the cytokine response in HS skin, we observed significant responses for stimulation of type II IFN (i.e., IFN-γ; *P* = 5.9 × 10^–5^) and IL-36 (*P* = 9.3 × 10^–4^) in HS lesional skin, whereas the effect of Th2 response (i.e., IL-4), IL-17A, or TNF stimulation was absent in HS skin (Figure 1C). These data demonstrate lack of a dominant Th cytokine axis in HS, in contrast to AD (Th2) or psoriasis (Th17). To address the unique inflammatory responses in HS, we compared HS with either psoriasis or AD and found that the most prominent genes unique to HS included genes encoding immunoglobulins (Figure 1D). Using bulk RNA-Seq data from HS skin, we interrogated for cell type–specific signatures. For HS skin the top 3 cell signatures were assigned to B cells (*P* < 1 × 10^–40^), followed by various T cell populations, including Th2, and CD4^+^ and CD8^+^ effector memory cells (*P* < 1 × 10^–12^) (Figure 1E). In contrast, cell type signatures in blood included CD4^+^ naive cells (*P* < 1 × 10^–20^), Th17 cells (*P* < 1 × 10^–15^), and Th2 cells (*P* < 1 × 10^–12^) (Figure 1E). Biological processes enriched among increased DEGs in HS skin included immune response (adjusted *P* = 7.64 × 10^–84^), regulation of immune response (adjusted *P* = 8.26 × 10^–82^), complement activation (adjusted *P* = 2.09 × 10^–56^), Fc-gamma receptor signaling pathway (FDR = 8.86 × 10^–41^), innate immune response (FDR = 4.92 × 10^–33^), B cell receptor signaling (FDR = 2.32 × 10^–23^), and neutrophil chemotaxis (FDR 8.75 × 10^–11^). Biological processes enriched among decreased DEGs included PPAR signaling pathway (adjusted *P* = 3.17 × 10^–7^) and steroid biosynthesis (adjusted *P* = 0.008). KEGG pathways among decreased DEGs included cholesterol biosynthetic process (adjusted *P* = 9.64 × 10^–11^) and lipid metabolic process (adjusted *P* = 5.5 × 10^–4^) (Figure 1F).

### Single-cell RNA-sequencing outlines the cellular composition of HS.

**Figure 2:**
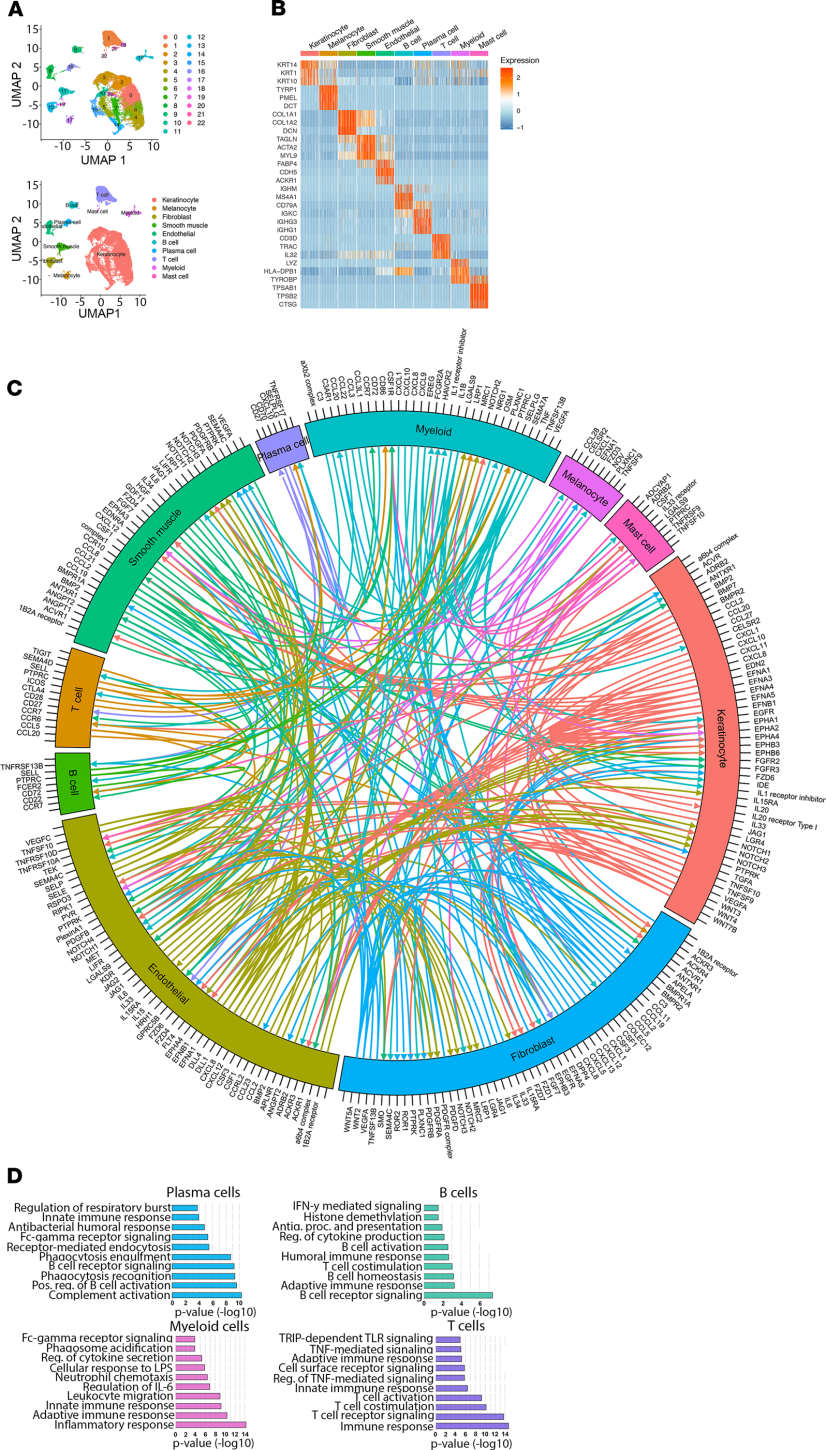
scRNA-Seq helps characterize the inflammatory cell composition of HS. scRNA-sequencing (scRNA-Seq) was performed on skin cells isolated from patients with moderate-to-severe HS undergoing surgical excisions (*n* = 9). Information on 30,636 cells across 23 cellular clusters representing 10 cellular subsets (**A**). Heatmap of the top 3 transcripts in each cluster showed clear demarcation between different clusters (**B**). Cell-cell receptor-ligand communication between inflammatory infiltrate and stromal tissues for the top 200 receptor-ligand pairs (**C**). Enriched biological categories among genes expressed in the 4 major inflammatory cell clusters (**D**).

**Figure 3:**
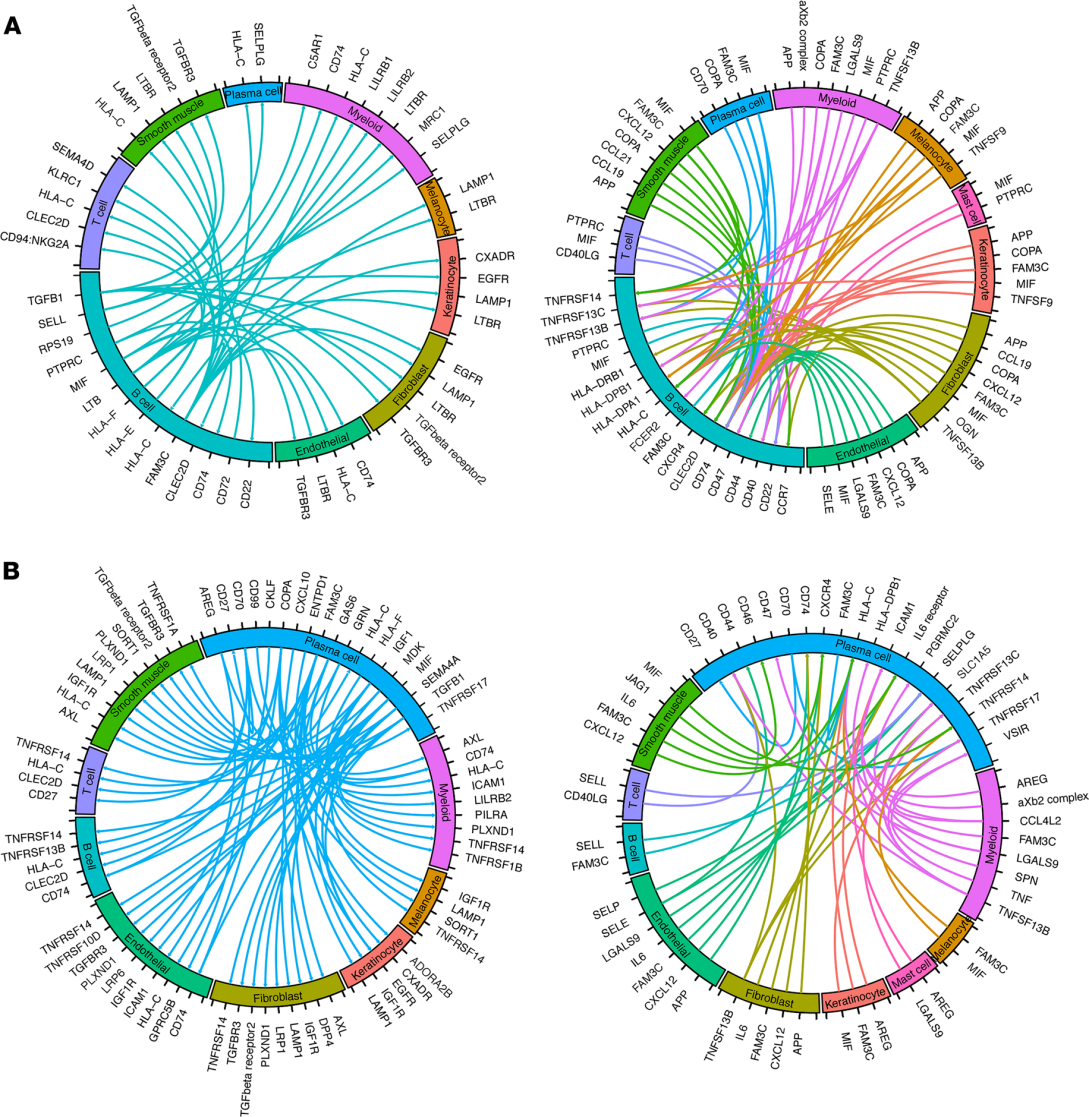
Interactions of B cells and plasma cells with HS microenvironment. Data from single-cell sequencing were used to map receptor-ligand interactions from and to B cells (**A**) and to and from plasma cells (**B**) (*n* = 9).

Single-cell RNA-sequencing (scRNA-Seq) was performed on cells isolated from 9 excisional samples from patients with severe HS. We collected 30,636 cells with a median 1974 genes and a median 7342 transcripts. We did unsupervised clustering analysis and grouped the cells into 22 clusters (Figure 2A), which were further annotated as 10 cell types including keratinocytes, melanocytes, fibroblasts, smooth muscle cells, endothelial cells, B cells, plasma cells, T cells, myeloid cells, and mast cells (Figure 2A). Three representative signature genes for each cell type are shown in the heatmap (Figure 2B). To learn cell-cell communication between the cell types, we performed CellPhoneDB ligand-receptor analysis and plotted the top ranked 200 pairs in a Circos plot (Figure 2C). We also show the connection linked from or to B cells and plasma cells (Figure 3, A and B). The ligand-receptor analysis demonstrated extensive interactions between all the major cell subsets in HS, including interactions of B cells and plasma cells with stromal tissue cells and other immune cell components. Notable interactions include members of the Notch signaling pathway with endothelial and keratinocyte cell populations, keratinocyte-derived CCL20 with T cells, and interaction of IFN-γ with various stromal cell clusters (Supplemental Table 4). We identified both *IL17A*- and *IFNG*-expressing T cells in HS skin (Supplemental Figure 4 and Supplemental Figure 5). Notably, *IL18*, a promoter of IFN-γ responses (16), was primarily derived from keratinocytes (17). We also performed enrichment analysis on the signature genes for each cell type, demonstrating enrichment of complement activation (*P* = 5.0 × 10^–11^), positive regulation of B cell activation (*P* = 2.8 × 10^–10^), and antibacterial humoral response (1.6 × 10^–5^) in the plasma cell population; B cell receptor signaling pathway (*P* = 4.1 × 10^–8^) and IFN-γ mediated signaling pathway (*P* = 0.03) in B cells; inflammatory response (*P* = 6.2 × 10^–15^) and neutrophil chemotaxis (*P* = 3.9 × 10^–7^) in the myeloid cell population; and T cell receptor signaling pathway (*P* = 1.8 × 10^–14^), T cell costimulation (*P* = 7.0 × 10^–11^), and regulation of TNF-mediated signaling (*P* = 8.4 × 10^–6^) in the T cell compartment (Figure 2D).

### Keratinocytes show heightened type II IFN and IL-36 responses.

**Figure 4:**
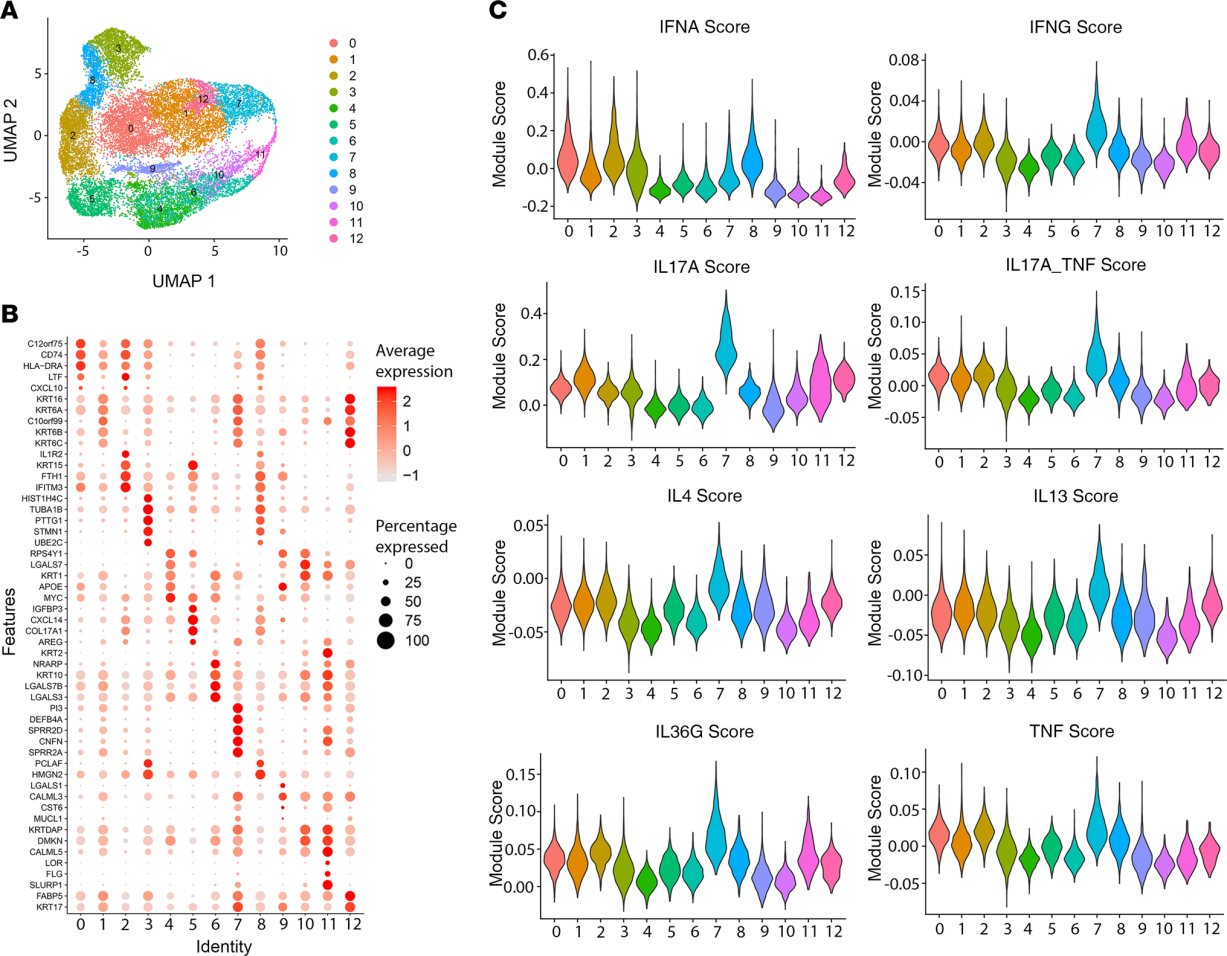
Inflammatory responses in HS keratinocytes have elevated type II IL-17, TNF, and IL-36 responses. Transcriptomic information was available on 20,587 keratinocytes from HS skin. These were divided into 13 clusters (**A**). A dot plot showing the top 3 markers for each cluster marked the defining genes for each cluster, although with some overlap between clusters (**B**). Transcriptomic cytokine responses from several proinflammatory cytokines were used to interrogate each keratinocyte for each particular inflammatory signature, with cluster 7 showing overall the highest and broadest inflammatory signal, but with different specific inflammatory responses having different cluster localization in HS keratinocytes (*n* = 9) (see Methods) (**C**).

**Figure 5:**
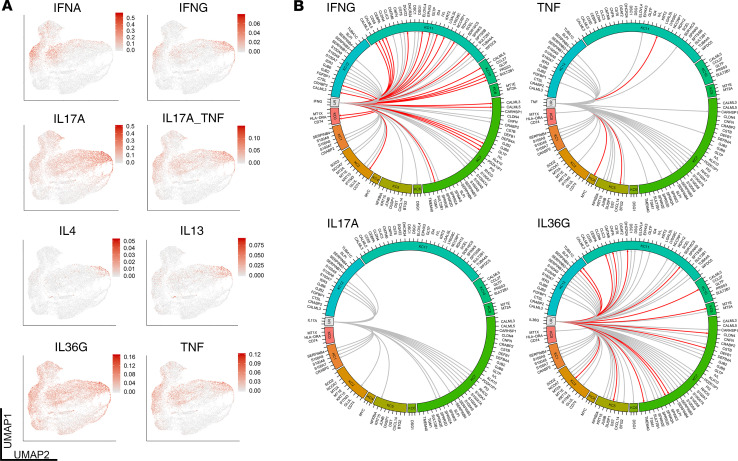
IFN-γ and IL-36 responses are the most prominent keratinocyte immune responses in HS skin. Specific cytokine responses were superimposed on the keratinocyte UMAP clusters to determine the distribution of key cytokine responses across different clusters (**A**). Circos plots were used to show the connection between the major inflammatory signals (lines) and the specificity (red line) to different keratinocyte clusters (clusters 0–12). Of these the IFN-γ and IL-36 responses had the highest degree of specificity (**B**) (*n* = 9).

To study the cytokine response in the largest population in our scRNA-Seq data, we performed subclustering on the keratinocytes and obtained 13 subclusters (Figure 4A). The top marker genes for each subcluster are shown in Figure 4B. We used a broad range of cytokine response signatures, as previously described by our group (15), to interrogate each keratinocyte subcluster for a particular inflammatory signature (Supplemental Table 5). A module score for each cytokine was calculated for each cell based on the expression of the signature genes induced by the cytokine. Then the module scores for each subcluster were plotted in Figure 4C. This demonstrates high inflammatory burden in keratinocytes in subcluster 7, particularly for IL-17 responses (adjusted *P* < 1 × 10^–250^), IL-36 responses (adjusted *P* = 6.4 × 10^–77^), and TNF responses (adjusted *P* = 2.7 × 10^–9^). This cluster was characterized by high expression of antimicrobial genes, such as *DEFB4A*, *S100A7A*, and *IL36G*. In contrast, subclusters 0, 1, 2, 3, and 8 showed enrichment for both type I and type II IFN responses (adjusted *P* < 1 × 10^–250^), with IFN-γ additionally showing an enriched response in subcluster 7 (adjusted *P* < 1 × 10^–250^). The distribution of each cytokine response can also be observed by plotting the module scores on the uniform manifold approximation and projection (UMAP) (Figure 5A). To address the specificity of each signal, we focused on the 4 main responses: IFN-γ, TNF, IL-17A, and IL-36. Of these, unique cytokine responses were most abundant for IFN-γ, followed by IL-36 and TNF (red lines) (Figure 5B), whereas nonspecific and overlapping responses were observed for IL-17A and to a lesser extent for the other cytokine responses (gray lines, Figure 5B). These data demonstrate that only a proportion of keratinocytes are responding to inflammatory stimuli in HS and that these responses are dominated by IFN-γ, followed by IL-36G and TNF, with lesser contribution by IL-17A.

### B cells and plasma cells predominate in HS skin lesions.

**Figure 6:**
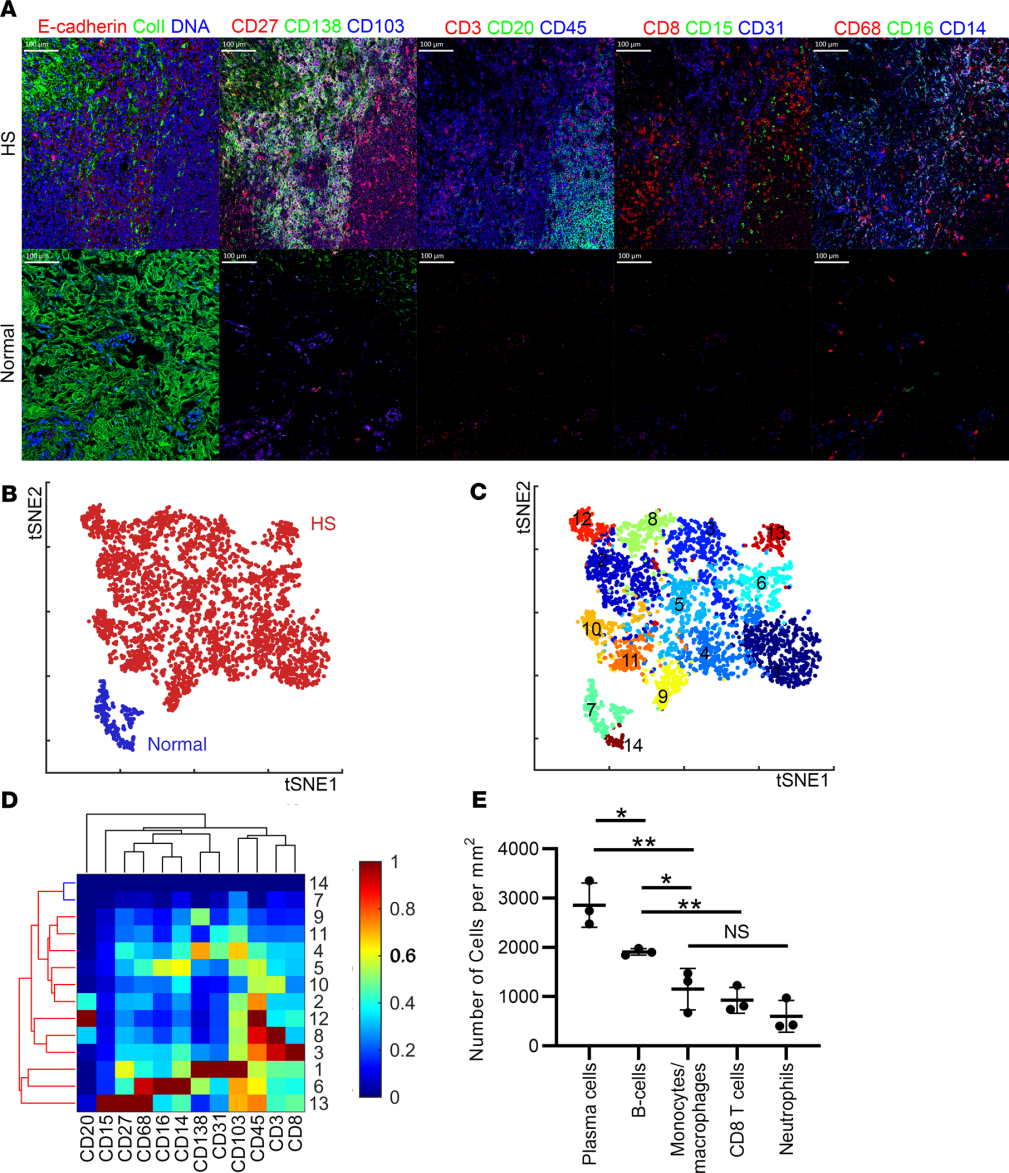
B and plasma cells are the dominant infiltrating leukocytes in HS. Analysis of the CyTOF data by t-distributed stochastic neighbor embedding (t-SNE) dimensionality reduction demonstrated clear separation between HS and normal skin (**A**) (scale bar: 100 μm), with the staining forming 14 distinct Phenograph clusters, of which only 2 were found in normal skin (**B** and **C**). Heatmap showing marker expression of each cluster (**D**). Quantification of the different subsets based on surface markers (**E**) (*n* = 3, Student’s *t* test, **P < 0.01; *P < 0.05; NS, nonsignificant).

To determine whether the transcriptomic signatures align with the cellular infiltrates in HS, we used CyTOF imaging to identify and quantify infiltrating leukocytes. HS skin had a marked increase in leukocyte infiltration compared with normal skin (Figure 6A). When visualized using the dimensional reduction tool t-SNE, there was complete separation between the leukocyte clustering in HS skin compared with normal skin, with 12 distinct clusters being observed in HS and 2 clusters in normal (Figure 6B). Markers characterizing each cluster are shown (Figure 6, C and D), with plasma cells being the predominant infiltrating leukocyte population in HS skin, followed by B cells, monocyte/macrophages, CD8^+^ T cells, and neutrophils (Figure 6E). These results demonstrate that plasma cells and B cells constitute the dominant infiltrating leukocyte population in HS. B cell and plasma cell subsets and associated gene transcripts are shown in Supplemental Figure 6.

### HS skin shows increased immunoglobulin production and diversity accompanied by complement activation.

**Figure 7:**
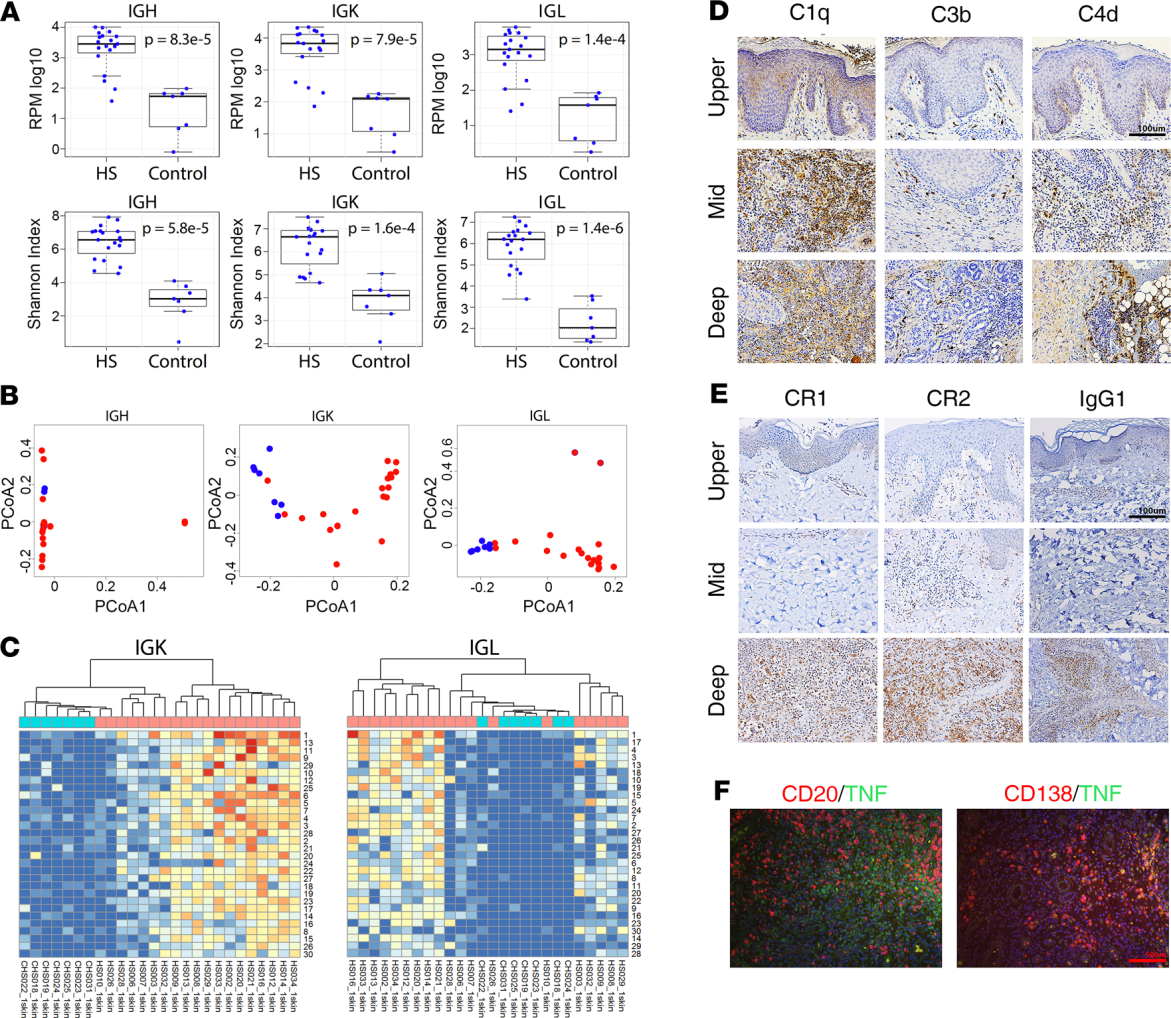
Increased immunoglobulin production and antibody diversity in HS skin and complement activation. Box-and-whisker plots of BCR CDR3 expressions (**A**). The *y* axis shows normalized log-transformed BCR CDR3 expression. The *x* axis represents patient group. In all cases there were more BCR CDR3 sequences detected in HS skin compared with control healthy skin. Box-and-whisker plots of BCR gene segment expression. The *y* axis shows normalized log-transformed BCR gene segment expression. The *x* axis represents patient group. The Shannon diversity index for BCR CDR3 gene segment is plotted on the *y* axis. The *x* axis represents patient group. HS skin had a significantly more diverse BCR repertoire (**B**). Beta diversity–based principal coordinates analysis (PCoA) of BCR CDR3 sequences. Sample matrix was generated using Jaccard dissimilarities, and respective profiles were compared by PCoA. Each color represents 1 patient group, HS (red) and control (blue). This analysis revealed clear separation for κ and λ light chains but not Ig heavy chain (**C**) Hierarchical clustering of expressed TCR V/J gene segment expression. Heatmaps by clonal abundance across sample sets. Note good separation of HS from controls based upon clonal abundances in BCR κ and λ repertoires. Components of the complement pathway (C1q) and breakdown products of activated complement components (C3b, C4d) were increased in HS skin, particularly in the deeper layers of the skin (*n* = 3) (scale bar: 100 μm) (**D**). Complement receptors, CR1 and CR2, were increased in the deeper layers of HS, along with IgG1 immune complex deposition (*n* = 3) (scale bar: 100 μm) (**E**). Immunofluorescence of B cells (CD20) and plasma cells (CD138) showed primary localization of TNF to the plasma cell population in HS skin (*n* = 3) (scale bar: 50 μm) (**F**). For **A** and **B**, the bold vertical line represents the median, and the upper and lower limits of the box represent the interquartile range (IQR). The whiskers represent 1.5× IQR.

To characterize the HS immune repertoire, B cell receptor (BCR) and TCR gene segments and complementarity determining region 3–encoding (CDR3-encoding) sequences were mined from RNA-Seq data sets obtained from skin and blood of HS patients and healthy controls. This analysis revealed that based upon BCR CDR3 sequences and BCR gene segment abundances, there was a significant increase in the frequency of B cells in HS skin (*P* = 7.8 × 10^–5^, 6.7 × 10^–5^, 9.8 × 10^–5^ and *P* = 8.3 × 10^–5^, 7.9 × 10^–5^, 1.4 × 10^–4^ for BCR IgH, κ, λ clones per million and BCR IgH, κ, λ gene segment reads per million, respectively) (Figure 7A). Next, to evaluate if there were any qualitative changes in the repertoire, BCR CDR3 alpha diversity was assessed by calculating the Shannon index (Figure 7A). This analysis revealed that the HS BCR repertoire was significantly more diverse than that of control skin (*P* = 5.8 × 10^–5^, 1.6 × 10^–4^, and 1.4 × 10^–6^ BCR IgH, κ, λ clone Shannon diversity, respectively). To further evaluate whether the cutaneous B cell repertoire was qualitatively altered in the setting of HS, Jaccard distances were calculated as a measure of dissimilarity. Principal coordinates analysis (PCoA) plots of the beta diversity measures indicated good segregation of samples by disease status for BCR κ and λ light chain (Figure 7B). There was no separation by disease status when this analysis was performed for IgH CDR3 sequences. Thus, at least at the level of the BCR light chain gene rearrangements, there were clear qualitative and quantitative differences in the B cell repertoire of patients with HS. To explore this finding further at the level of individual B cell clones, heatmaps were constructed from data on clone frequencies across HS and healthy control skin. Similar to the PCoA plots, the frequencies of clonal abundance at the level of BCR κ and λ CDR3s across subjects was different enough to separate HS from healthy controls (Figure 7C). To assess deposition of IgGs and complement activation in HS, we stained for components and breakdown products of the complement pathway. This showed prominent protein expression of C1q, C3b, and C4d in HS skin, particularly in the deeper layers of the skin (Figure 7D). Complement receptors CR1 and CR2 were also increased in HS skin, along with IgG immune complex deposition (Figure 7E). To determine if this contributed to the proinflammatory environment in HS, we costained for TNF, the best characterized inflammatory mediator in HS, against B cells (CD20) and plasma cells (CD138). This showed nearly exclusive colocalization of TNF to plasma cells (Figure 7F). Less pronounced shifts in the mRNA expression of TCRs (*TRA*, *TRB*, *TRD*, *TRG*) was seen, in terms of both total reads and diversity (Supplemental Figure 3).

### BCR and Bruton’s tyrosine kinase signaling are potential therapeutic targets in HS.

**Figure 8:**
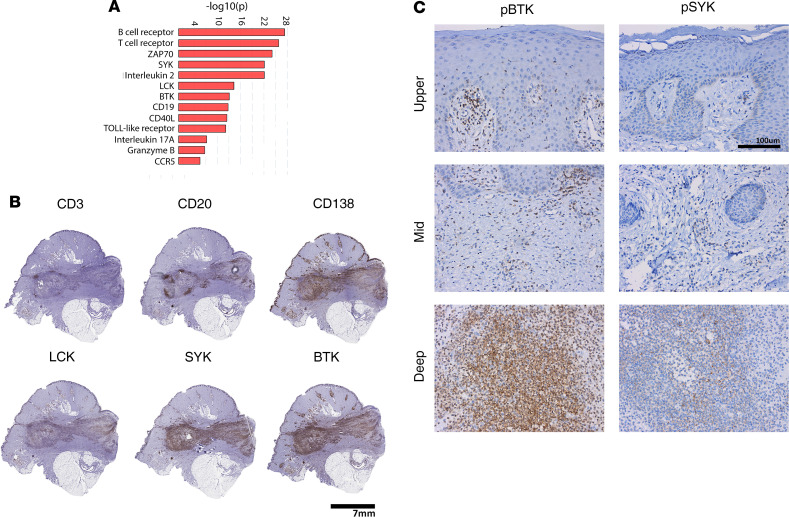
Enrichment and activation of B cell–associated signaling pathways in HS skin. Analysis of the signal transduction networks using literature-based networks (Genomatix-Pathway System, GePS) demonstrated enrichment for pathways involved in B cell signaling and activation (**A**). To confirm the nature of the inflammatory infiltrate in HS and the localization of components of the enriched signaling pathways, we performed IHC in an excisional biopsy for CD3, CD20, and CD138. Plasma cells were the predominant inflammatory infiltrate and most prominent in the deeper layers of the skin surrounding a deeper sinus tract (**A**), accompanied by increased expression of BTK, SYK, and LCK (**B**) (*n* = 3). Activation of key components of this signaling pathway was confirmed by IHC for both phospho-BTK and phospho-SYK (*n* = 3) (**C**).

**Figure 9:**
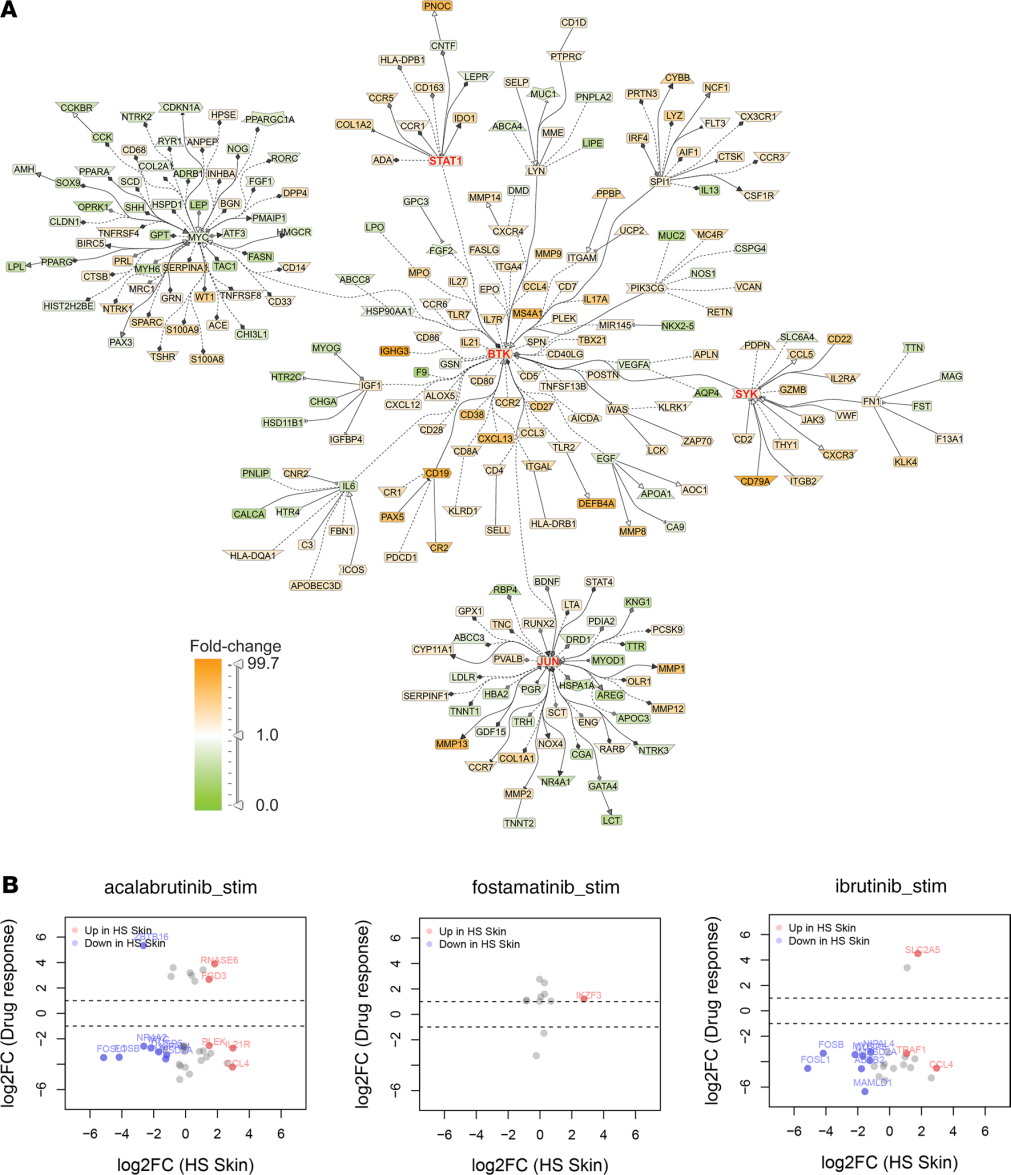
B cell receptor signaling is central to HS transcriptomic changes and a potential therapeutic target in HS. Outline of the GePS network in HS skin centered on critical inflammatory nodes, including BTK, SYK, JUN, and STAT1 signaling (red/brown indicating increased expression and green indicating decreased expression) (**A**). Overlap between gene expression in activated B cells (IgG/IgM stimulated) treated with the BTK inhibitors acalabrutinib and ibrutinib and the SYK inhibitor fostamatinib (**B**) (*n* = 3).

To validate our transcriptomic findings, we performed IHC of excisional HS tissue of longstanding duration and with prominent sinus formation. This showed positive staining for CD3^+^ T cells, CD20^+^ B cells, and CD138^+^ plasma cells mostly concentrated in the deeper aspect of the biopsy, around a sinus tract (Figure 8A). Analysis of the signal transduction network using literature-based networks (GePS) demonstrated enrichment for BCR signaling (*P* < 1 × 10^–25^), spleen tyrosine kinase (SYK) signaling (*P* < 1 × 10^–22^), and Bruton’s tyrosine kinase (BTK) signaling (*P* < 1 × 10^–13^), with enriched signals for IL-17A (*P* < 1 × 10^–7^), granzyme B (*P* < 1 × 10^–7^), and CCR5 (*P* < 1 × 10^–4^) (Figure 8B). Activation of these signaling pathways was validated by IHC for phospho-BTK and phospho-SYK (Figure 8C). Network analysis showed prominent clustering of increased HS DEGs around BTK and SYK nodes (Figure 9A). To determine therapeutic potential of these targets in HS, we used IgG/IgM-stimulated B cells, treated with the BTK inhibitors acalabrutinib and ibrutinib, or the SYK inhibitor fostamatinib, and determined transcriptomic overlap and responses against DEGs in HS skin. The monocyte chemokine *CCL4* was one of the most highly expressed chemokines in HS skin, with its mRNA expression increased by 7.7-fold (FDR = 4.1 × 10^–3^). Its expression was suppressed 19-fold by acalabrutinib, adjusted *P* = 5.6 × 10^–5^, and 22.8-fold by ibrutinib, adjusted *P* = 2.2 × 10^–3^ (Supplemental Table 6). Less pronounced overlap was seen for the SYK inhibitor fostamatinib (Figure 9B).

## Discussion

The etiology of HS is still incompletely understood but appears to be complex, with multiple factors contributing to its pathogenesis, including obesity, sex, hormonal factors (5), dysregulated microbiota (18, 19), and genetics (6). In contrast to many other chronic inflammatory diseases, no genome-wide association studies have been performed in HS. However, studies on autosomal dominant forms of HS have identified mutations in genes belonging to the gamma-secretase complex, involved in regulation of Notch signaling activity (6), although in sporadic HS, these mutations seem to represent only a small minority of HS cases (7). One of the hallmark features of HS is the presence of deeply invasive epithelial tendrils that form tracts and keratin-filled cysts. Notably, in mouse skin, in the absence of gamma-secretase function, there is conversion of hair follicles to epidermal cysts with irregular ingrowths (20), suggesting that abnormal activity of gamma-secretases, and Notch signaling, may drive this feature of HS, although in this model no inflammation was noted (20). Whether the inflammatory response in HS is driving the ingrowth of epithelial tendrils or, conversely, tendril growth followed by rupture and release of keratinized and bacterial contents from the cysts and tracts is responsible for the inflammation is still unknown. While none of the NOTCH, NOTCH ligands, or gamma-secretase genes (*NCSTN*, *PSENEN*, and *PSEN1*) were differentially expressed in our data set, it is possible that our data did not capture changes in gamma-secretase or Notch signaling, as our study was focused on patients with chronic, established inflammatory disease. However, the early sequence of events in HS will need to be addressed in future studies.

Several studies have implicated complement activation in the pathogenesis of HS. This includes studies showing elevated C5a (8), which is a breakdown product of complement pathway activation and a major neutrophil chemoattractant, in both blood and skin of HS patients (8). Neutrophils are prominent in HS (9), and a recent study demonstrated increased levels of neutrophils undergoing NETosis in both blood and skin of HS patients, with the amount of NETs disgorged in HS skin correlating with disease severity (10). Furthermore, this same study identified increased levels of autoantibodies recognizing citrullinated peptides and NET antigens (10). A recently published clinical trial using an IgG5 monoclonal antibody that selectively binds to C5a and blocks its biological activity demonstrated modest clinical efficacy in HS (21).

Transcriptomic profiling of HS skin has been performed by several groups, although these studies have been limited to relatively low-resolution microarray-based studies (11, 13, 22). These studies have shown increased number of infiltrating CD4^+^ T cells secreting IL-17 and IFN-γ and increased mRNA expression of *IL17A* and *IFNG* in HS skin, accompanied by increased IL-17A CD4^+^ T cells in HS blood, but unchanged frequency of IFN-γ–secreting cells (11). These studies have suggested IL-17–centric pathogenesis, supported by the role of IL-17A in psoriasis as one of the most highly enriched biological processes in HS skin, followed by interferon signaling (11), and are consistent with findings from our own group that showed increased protein levels of IL-17A and to a lesser extent IFN-γ in lesional HS skin (12). Furthermore, keratinocyte-derived IL-6 and IL-1β have been implicated in HS pathogenesis (11). Our data did not detect increased mRNA expression of *IL1B* in HS skin, and *IL6* was decreased by about 4-fold (adjusted *P* = 2.2 × 10^–3^) (Supplemental Table 2). Instead, we found evidence for increased expression of *IL36A* and *IL36G* (Figure 1). In this context, case reports targeting the IL-1 axis in HS have provided mixed results, with some showing modest improvement (23, 24) and others showing no improvement (25). No clinical trial data exist on the use of anti–IL-6 agents in HS, but one study noted development of HS in a patient undergoing the anti–IL-6 treatment tocilizumab (26).

Other inflammatory mediators implicated in HS include the type I IFNs, based primarily on the expression of IFN response genes, such as *MX1*, *CXCL10*, and *IFI27*. The expression of these was proposed to be driven by NETs interacting with infiltrating pDCs in lesional HS skin (10). In agreement with this study, we found evidence for both type I and type II IFN responses in our data set, most prominently in 4 of the scRNA-Seq keratinocyte clusters, which notably did not overlap with other clusters having pronounced IL-17 and TNF responses (Figure 4 and Figure 5). Of note, we were unable to detect pDCs in the scRNA-Seq data, and there was no increase in the mRNA expression of any of the type I IFN genes or the pDC markers *CD123* or *CD303*. In contrast, mRNA expression of *IFNG* was increased by about 2.6-fold, and *CXCL10* increased by about 2.7-fold, suggesting that IFN-γ might have a greater contribution to the IFN response in HS than the type I IFNs.

Our data identified B cells, and in particular plasma cells, as a potential therapeutic target in HS. Strikingly, TNF expression, the target of the only approved biologic treatment in HS (27), was localized to CD138^+^ plasma cells (Figure 7G). Successful use of B cell targeting in HS has been reported with the anti-CD20 agent rituximab in a patient undergoing treatment for an unrelated immune disorder (28). An ex vivo explant study of 10 patients in which skin explants were treated with rituximab demonstrated significant decrease in secretion of several proinflammatory mediators (12). However, anti-CD20 treatment would be unlikely to target plasma cells, as CD20 is downregulated in plasma cells with CD20 detected only on a small minority of plasma cells (29). An alternative approach to target plasma cells is to block active signaling pathways in these cells. The most enriched pathways in HS involved B cell signaling, including BCR signaling, SYK, LCK, and BTK (Figure 8A), and these pathways correlated with B cell (CD3) and plasma cell (CD138) infiltration in HS skin but not T cell (CD3) (Figure 8B). Notably, drugs targeting BTK have been shown to prevent plasma cell generation (30), and SYK has been shown to play a critical role for B cell antibody responses, memory B cell survival (31), and plasma cell expansion (32). While our therapeutic data were limited to B cells, they showed decreased B cell expression of several proinflammatory mediators that overlapped with HS skin, including CCL4, and this was more pronounced with the BTK inhibitors acalabrutinib and ibrutinib than the SYK inhibitor fostamatinib (Figure 9B). Consistent with an active role of B cells in HS pathogenesis is the observation that these cells are prominent in early HS lesions and are the major immune cell population attenuated with anti-TNF treatment (33).

In summary, while our data have some limitations, including analysis of mostly one ethnicity and lack of perilesional and matched unaffected skin from patients, we provide a comprehensive overview of the inflammatory pathogenesis of HS and help tie together previously discordant data. As there are no animal models of this disease, our data, through identification of potentially novel therapeutic signaling pathways, provide preclinical evidence that should accelerate the path toward clinical trials targeting either BTK or SYK in chronic, moderate-to-severe HS.

## Methods

### Single-cell RNA-sequencing.

Generation of single-cell suspensions for scRNA-Seq was performed as follows: lesional HS tissue was obtained from excisional samples from patients with severe HS. Samples were incubated overnight in 0.4% dispase (Life Technologies, Thermo Fisher Scientific) in Hank’s Balanced Saline Solution (Gibco, Thermo Fisher Scientific) at 4°C. Epidermis and dermis were separated. Epidermis was digested in 0.25% Trypsin-EDTA (Gibco, Thermo Fisher Scientific) with 10 U/mL DNase I (Thermo Fisher Scientific) for 1 hour at 37°C, quenched with FBS (Atlanta Biologicals), and strained through a 70 μM mesh. Dermis was minced, digested in 0.2% Collagenase II (Life Technologies, Thermo Fisher Scientific) and 0.2% Collagenase V (MilliporeSigma) in plain medium for 1.5 hours at 37°C, and strained through a 70 μM mesh. Epidermal and dermal cells were recombined, and libraries were constructed by the University of Michigan Advanced Genomics Core on the 10x Genomics Chromium system. Libraries were then sequenced on the Illumina NovaSeq 6000 sequencer to generate 151 bp paired-end reads. Data processing, including quality control, read alignment, and gene quantification, was conducted using the 10x Genomics Cell Ranger software. Seurat was used for normalization, data integration, and clustering analysis (34). Clustered cells were mapped to corresponding cell types by matching cell cluster gene signatures with putative cell type–specific markers.

### RNA-sequencing.

Skin biopsies of 4 mm in diameter were taken, placed in tubes with RNA*later* (Invitrogen, Thermo Fisher Scientific), stored overnight at 4°C, and subsequently stored at –80°C until further processing. RNA isolation and sequencing were performed using the Illumina NextSeq platform and sequencing protocols as previously described (35). For RNA-Seq analyses, adapter trimming and quality control were conducted on the raw sequence reads. The paired-end reads were mapped using STAR (36) to human build GRCh37, and only uniquely mapped reads were used for subsequent analysis. RNA-Seq data from psoriasis and AD were obtained (37). Gene expression levels were quantified (GENCODE v24 was used as reference) and normalized by HTSeq (38) and DESeq2 (39), respectively. Negative binomial model in DESeq2 were used to conduct differential expression analysis.

### TCR/BCR analyses.

MiXCR software was used to extract TCR and BCR CDR3 sequences from RNA-Seq data. Analysis was performed with the “-p rna-seq” option recommended for analysis of RNA-Seq data (40, 41). Data visualization and TCR repertoire comparison were performed in R (42, 43). The CDR3 region was defined according to the International ImMunoGeneTics (IMGT) nomenclature. Likewise, gene names of V and J regions were defined according to the IMGT name nomenclature for TCRs of mice as previously described (44, 45). Clone abundances across samples were plotted using the “pheatmap” R package (https://cran.r-project.org/web/packages/pheatmap/index.html). The heatmap was constructed using Euclidean distance with complete linkage on centered data using the top clones showing greatest variance. Alpha diversity was assessed by calculating the Shannon diversity index using the vegan software package in R (https://cran.r-project.org/web/packages/vegan/index.html). The Shannon diversity index quantifies diversity through the incorporation of both evenness and richness, with higher values representing more even populations of TCR- or BCR-rich samples. The differences in beta diversity were assessed based on Jaccard distances calculated using the vegan software package in R. To visualize the Jaccard dissimilarities between the samples, PCoA using R package ape was performed.

### CyTOF imaging.

Formalin-fixed, paraffin-embedded tissue slides obtained from HS patients and healthy controls were heated for 2 hours at 60°C, deparaffinized, and rehydrated. Slides were placed in pH 9 Tris/EDTA antigen retrieval buffer and heated at 96°C for 30 minutes. After cooling, slides were blocked in 3% BSA and stained with a cocktail of metal-tagged antibodies overnight at 4°C, including CD14 (EPR3653, Abcam), CD16 (EPR16784, Abcam), CD68 (KP1, BioLegend), CD15 (W6D3, Fluidigm), CD31 (JC/70A, Novus), CD45 (2B11, Novus), E-Cadherin (24E10, Fluidigm), CD20 (L26, Novus), CD8 (C8/144B, Fluidigm), collagen (polyclonal, Fluidigm), CD27 (EPR8569, Abcam), CD103 [EPR4166(2), Abcam], CD138 (MI15, BioLegend), and pan-actin (D18C11, Cell Signaling Technology). The slides were then washed with 0.2% Triton X-100 and stained with Intercalator-Ir (Fluidigm) for 30 minutes at room temperature in a hydration chamber. The stained tissue was ablated and raw data were acquired on the Hyperion Imaging System (Fluidigm).

### CyTOF imaging data analysis.

Multiplexed CyTOF imaging data were preprocessed using commercial acquisition software (Fluidigm) and converted to.TIFF images. These images were then segmented into individual cells using CellProfiler v3.1.8 for single-cell analysis. The t-SNE dimensionality reduction algorithm and the Phenograph unsupervised clustering algorithm were performed on 12 markers (CD20, CD15, CD27, CD68, CD16, CD14, CD138, CD31, CD103, CD45, CD3, and CD8) using HistoCAT v1.75 software. For t-SNE and Phenograph, the data were normalized to the 99th percentile. The heatmap shows *Z*-scored mean marker expression of each cluster. *P* values were computed using 2-tailed Student’s *t* tests assuming homoscedasticity.

### Immunohistochemistry.

Paraffin-embedded tissue sections from excisional biopsies from patients with hidradenitis and healthy control skin were heated at 60°C for 30 minutes, deparaffinized, and rehydrated. Slides were placed in pH 6 antigen retrieval buffer and heated at 125°C for 30 seconds in a pressure cooker water bath. After cooling, slides were treated with 3% H_2_O_2_ (5 minutes) and blocked using 10% goat serum (30 minutes). Overnight incubation (4°C) was performed using CR1 (CD35) (LifeSpan Biosciences, catalog LS-C675585, 5 μg/mL) and CR2 (CD21) (LifeSpan Biosciences, catalog LS-C167018, concentration 1:50). Staining done in antigen retrieval buffer at pH 9 included IgG1 (Abcam, catalog ab233885, 5 μg/mL), phospho-SYK (Cell Signaling Technology, catalog C87C1, 1:100), phospho-BTK (Invitrogen, Thermo Fisher Scientific, catalog 14-9015-82, 1:100), CD3 (Abcam, catalog ab17143, 1:10), C1q (LifeSpan Biosciences, catalog LS-B14993-100, 2 μg/mL), C3b (Abcam, catalog ab200999, 2 μg/mL), C4d (LifeSpan Biosciences, catalog LS-B3921-125, 2 μg/mL), LCK (LifeSpan Biosciences, catalog LS-B2049-50, 5 μg/mL), BTK (Sino Biological, catalog 10578-T44-50, 1 μg/mL), and SYK (Abcam, catalog ab40781, 1 μg/mL). Slides were then washed and treated with appropriate secondary antibodies, peroxidase (30 minutes), and diaminobenzidine substrate.

### Immunofluorescence.

Formalin-fixed, paraffin-embedded tissue slides obtained from patients and healthy controls were heated for 30 minutes at 60°C, deparaffinized, and rehydrated. Slides were placed in pH 9 antigen retrieval buffer and heated at 125°C for 30 seconds in a pressure cooker water bath. After cooling, slides were blocked using 10% donkey serum (30 minutes). Overnight coincubation (4°C) was then performed using anti–human TNF (Abcam, catalog ab6671), anti–human CD20 (Santa Cruz Biotechnology, catalog sc-393894), and anti–human CD138 (LifeSpan Biosciences, LS-B9360-50). Slides were then washed and treated with relative fluorescence-conjugated secondary antibodies (30 minutes). The secondary antibody used with anti–human TNF was Alexa Fluor 488 AffiniPure donkey anti–rabbit IgG (Jackson ImmunoResearch Laboratories, catalog 711-545-152). The secondary antibody used with anti–human CD20 and anti–human CD138 was Alexa Fluor 594 AffiniPure donkey anti-mouse IgG (Jackson ImmunoResearch Laboratories, catalog 715-585-151). Slides were prepared in mounting medium with 4′,6-diamidino-2-phenylindole (DAPI) (VECTASHIELD Antifade Mounting Medium with DAPI, H-1200, Vector Laboratories, Maravai LifeSciences). Images were acquired using a ZEISS Axioskop 2 microscope. Images presented are representative of at least 3 biologic replicates.

### B cell stimulations and inhibitor experiments.

Fostamatinib disodium, ibrutinib, and acalabrutinib in DMSO were purchased from Selleck Chemicals LLC. Lymphoprep (STEMCELL Technologies) was used to isolate the buffy coat from healthy donor volunteer blood, and the EasySep Human B Cell Isolation Kit (STEMCELL Technologies) was used to isolate B cells. Cells were incubated overnight at 37°C. Stimulation was performed with 5 μg/mL each of Goat F(ab′)_2_ Anti-Human IgM and Goat F(ab′)_2_ Anti-Human IgG (SouthernBiotech, catalog 2022-01 and 2042-01) in the presence of fostamatinib disodium (1 μM), ibrutinib (0.4 μM), acalabrutinib (1 μM), or DMSO control for 6 hours at 37°C. Viability assessed by trypan blue exclusion exceeded 90% for all conditions. Cells were washed in cold PBS, and RNA was isolated using the RNeasy Mini Kit (QIAGEN). Stranded mRNA libraries were prepared using the NEBNext Ultra II RNA Library Prep with poly(A) (New England Biolabs) and sequenced on the Illumina NovaSeq 6000 sequencer by the University of Michigan Advanced Genomics Core. Data were analyzed using the Scientific Data Analysis Platform (SciDAP) (Datirium) (46): Trim Galore RNA-Seq pipeline paired-end strand specific was used to trim adapters, map reads to GRCh38 (hg38), and quantify gene expression. DESeq (39) was used to perform differential expression analysis. Additional details on SciDAP pipelines are available at https://scidap.com/public/workflows

### Data availability.

Transcriptomic data sets can be accessed at the National Center for Biotechnology Information’s Gene Expression Omnibus database (GSE154775 and GSE154773).

### Statistics.

PCA was conducted using inverse-normalized expression levels of all detectable transcripts. FDR ≤ 10% and |log_2_ fold change| ≥ 1 were used to declare significance in differential expression analysis. Significantly regulated genes were analyzed by creating biological literature-based networks using software (GePS, version v3. 110621) (https://www.genomatix.de). The function-word level was used as the minimum evidence level parameter. We ran CellPhoneDB using all the cell types with the default parameters (47). For all single-group comparisons, if data passed normality test, we used 2-tailed Student’s *t* test. Otherwise data were analyzed using the Mann-Whitney *U* test. All data are representative of at least 2 independent experiments as detailed in the figure legends. A *P* value of less than or equal to 0.05 was considered significant. For all data related to the RNA-Seq analyses, a threshold of FDR ≤ 0.1 was used for significance.

### Study approval.

Twenty-two patients with moderate-to-severe HS undergoing surgery were recruited from the specialized HS outpatient clinic of the Department of Dermatology, Erasmus University Medical Center (Erasmus MC), Rotterdam, the Netherlands. Patients were off any topical or systemic treatment at least 2 weeks before enrollment. Biopsies from lesional skin and blood samples were collected during routine HS surgery. Ten healthy controls were recruited from patients undergoing surgery for skin cancer or abdominoplasty. All samples were obtained with written informed consent from the participants in accordance with Declaration of Helsinki principles. The study protocol was approved by the Institutional Review Board of the Erasmus MC (MEC-2013-337). The patient characteristics are listed in Supplemental Table 1.

## Author contributions

Design of study was performed by JEG, ASM, JJV, JMK, MDR, EM, RLM, and EPP; subject recruitment and sample collection were performed by KRVS, ARJVV, HHVDZ, PWH, JRCB, JHK, SW, and REB; conducting experiments and data acquirement were done by ACB, KRVS, EX, OP, KCC, SMR, CMY, CZ, XX, YJ, JK, and KCC; data and bioinformatic analyses were performed by LCT, FM, RW, MTP, MML, SMR, and CB; and interpretation of data and writing were performed by JEG, LCT, ACB, KCC, MML, FW, JMK, EM, RLM, and EPP.

## Supplementary Material

Supplemental data

Supplemental Table 2

Supplemental Table 3

Supplemental Table 4

Supplemental Table 5

Supplemental Table 6

## Version 1 08/27/2020

In-Press Preview

## Version 2 10/02/2020

Electronic publication
