# Supplementary material for: Contribution of plasma cells and B cells to hidradenitis suppurativa pathogenesis
Source: JCI Insight. 2020 Oct 2;5(19):e139930. doi: 10.1172/jci.insight.139930 (PMC7566715; doi:10.1172/jci.insight.139930)
Supplement: Supplemental data [file jciinsight-5-139930-s078.pdf]

SUPPLEMENTAL DATA

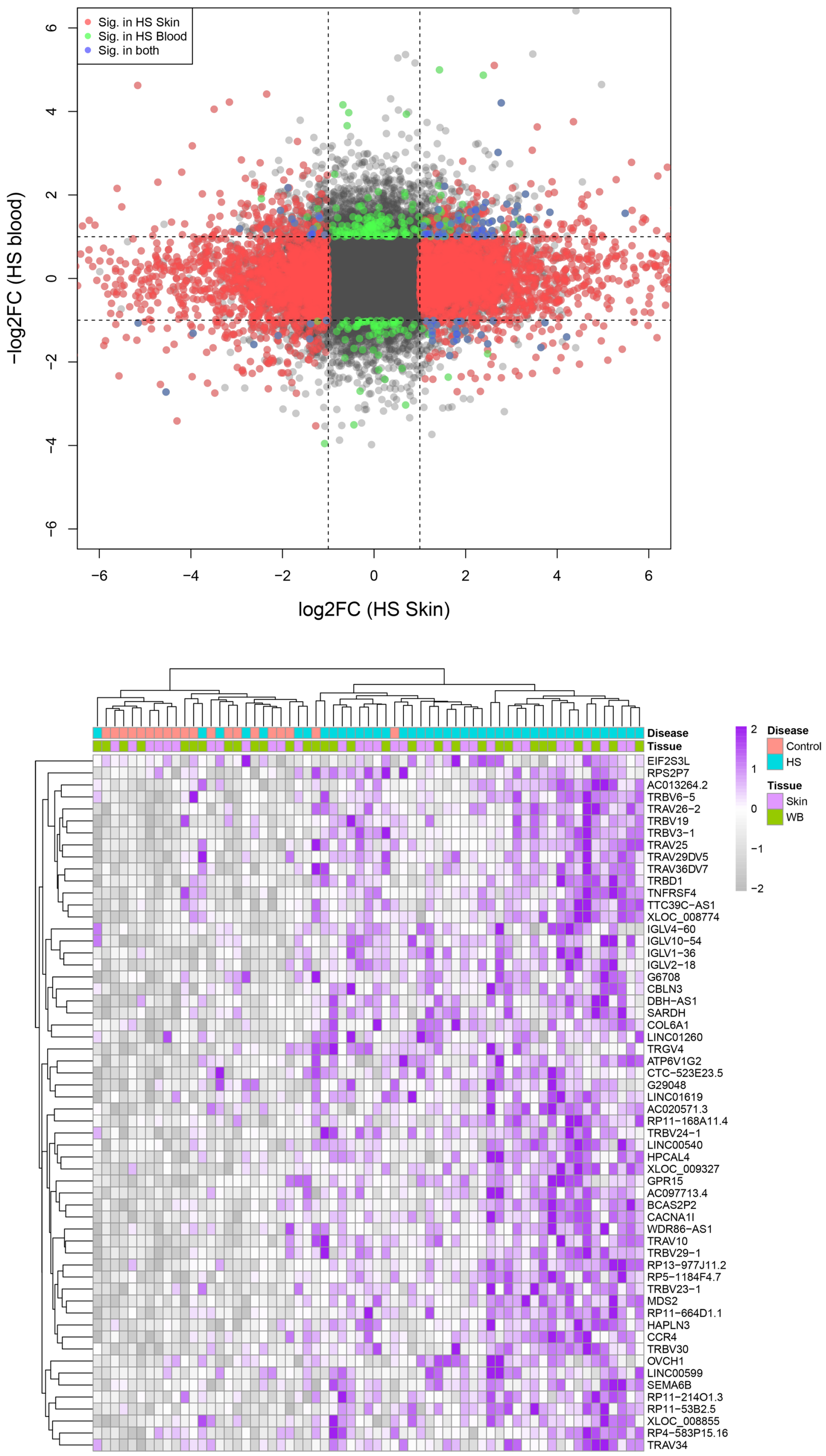

Supplemental Figure 1. Overlap between gene expression in HS skin and blood.

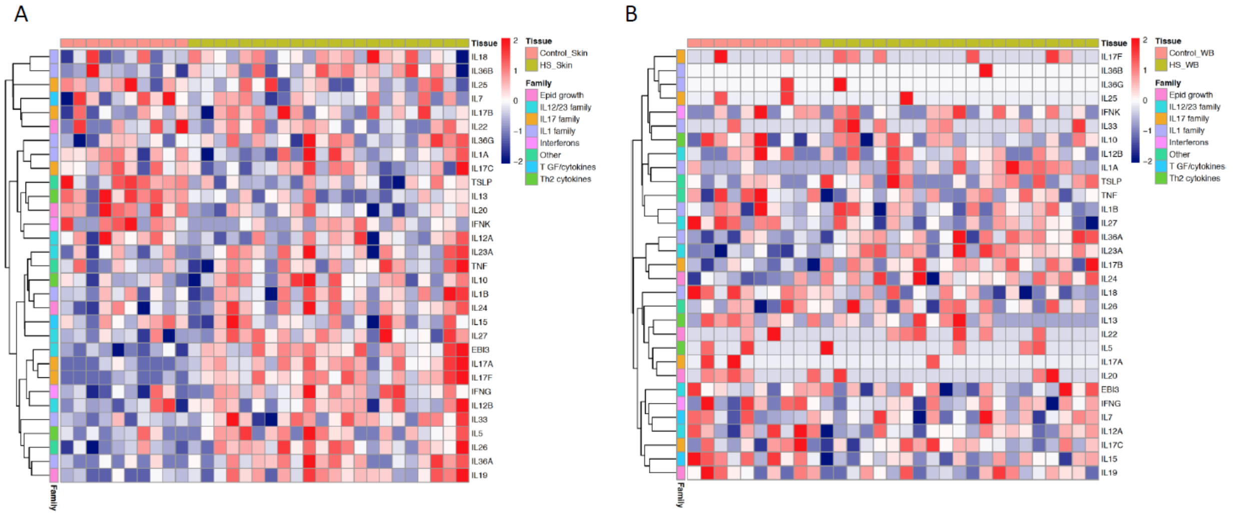

Supplemental Figure 2. Heatmap of cytokine gene expression in control and HS skin (left) and control and HS blood (right).

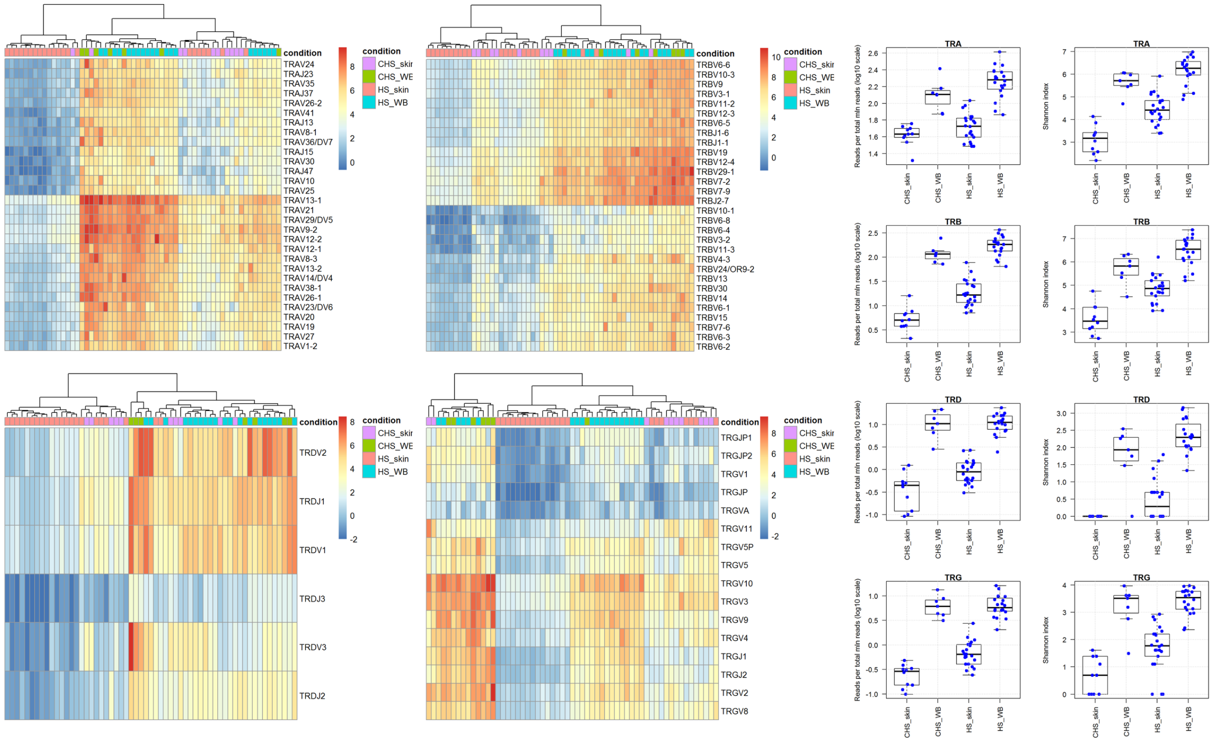

Supplemental Figure 3. T-cell receptor gene expression (alpha/beta, and gamma/delta) and diversity.

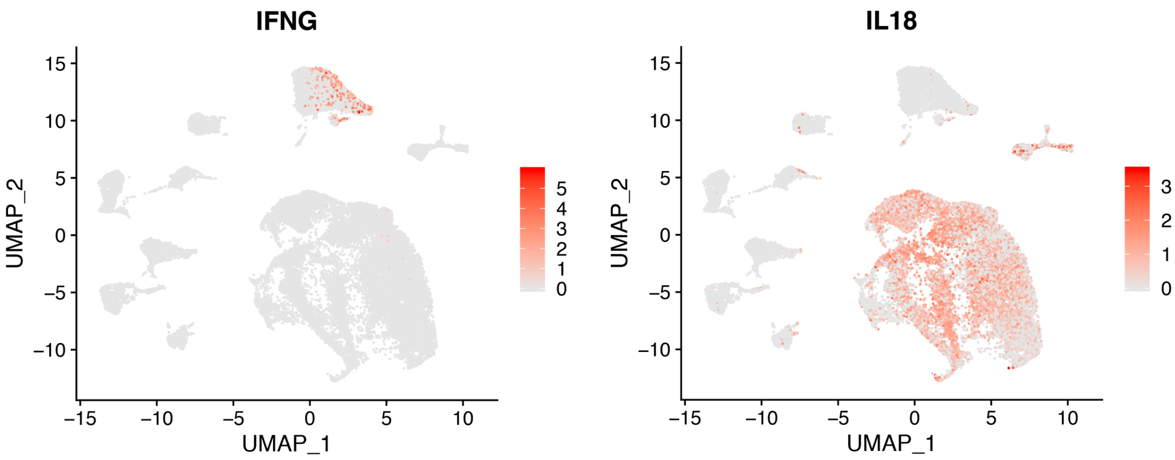

Supplemental Figure 4. ***IFNG*** **and** ***IL18*** **expression in HS.**

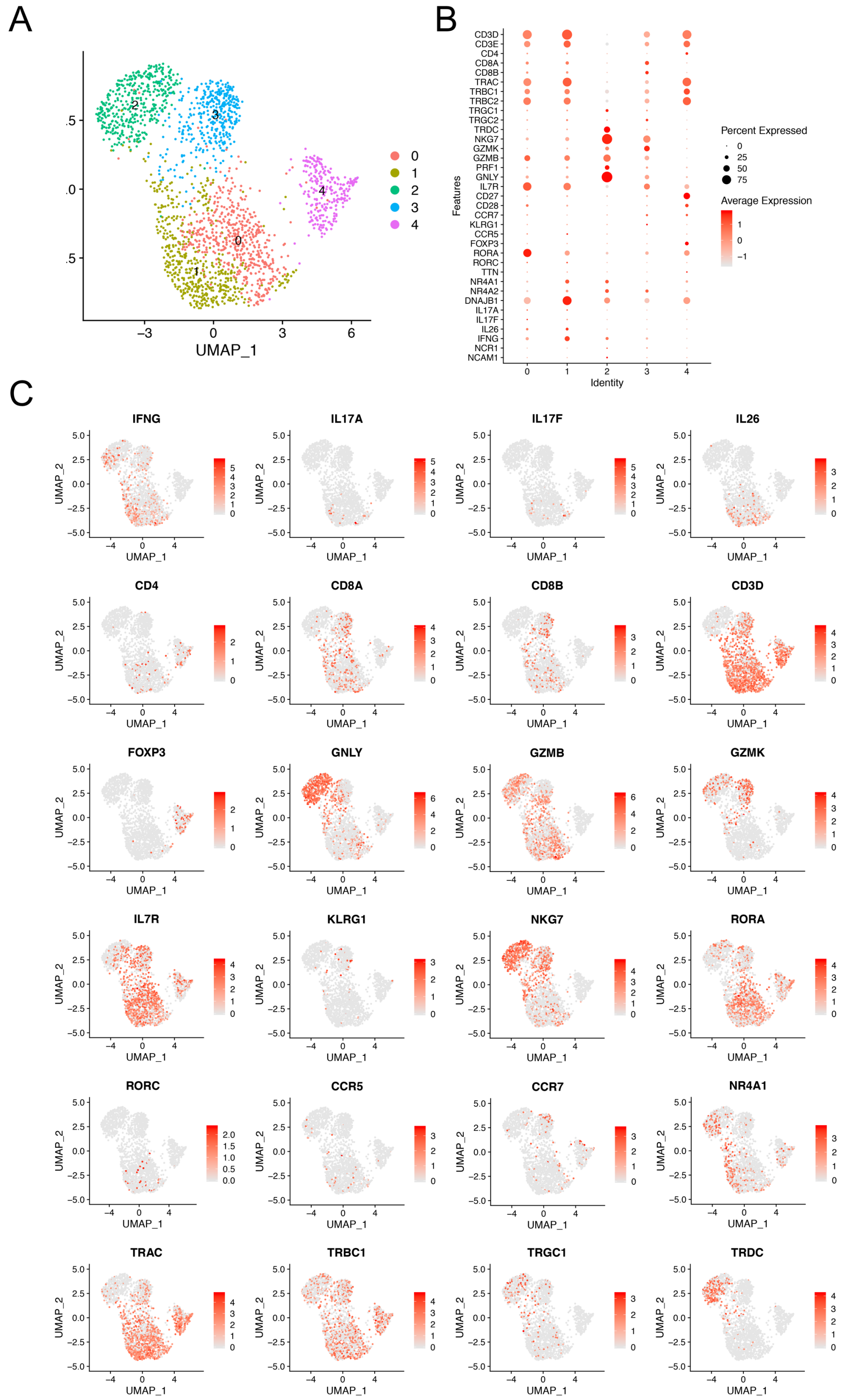

Supplemental Figure 5. T cell subsets in HS skin.

Subclustering of T-cells in HS skin (A). Marker genes for each of the 5 T cell subcluster (B). Expression of key T cell genes in UMAP clusters (C).

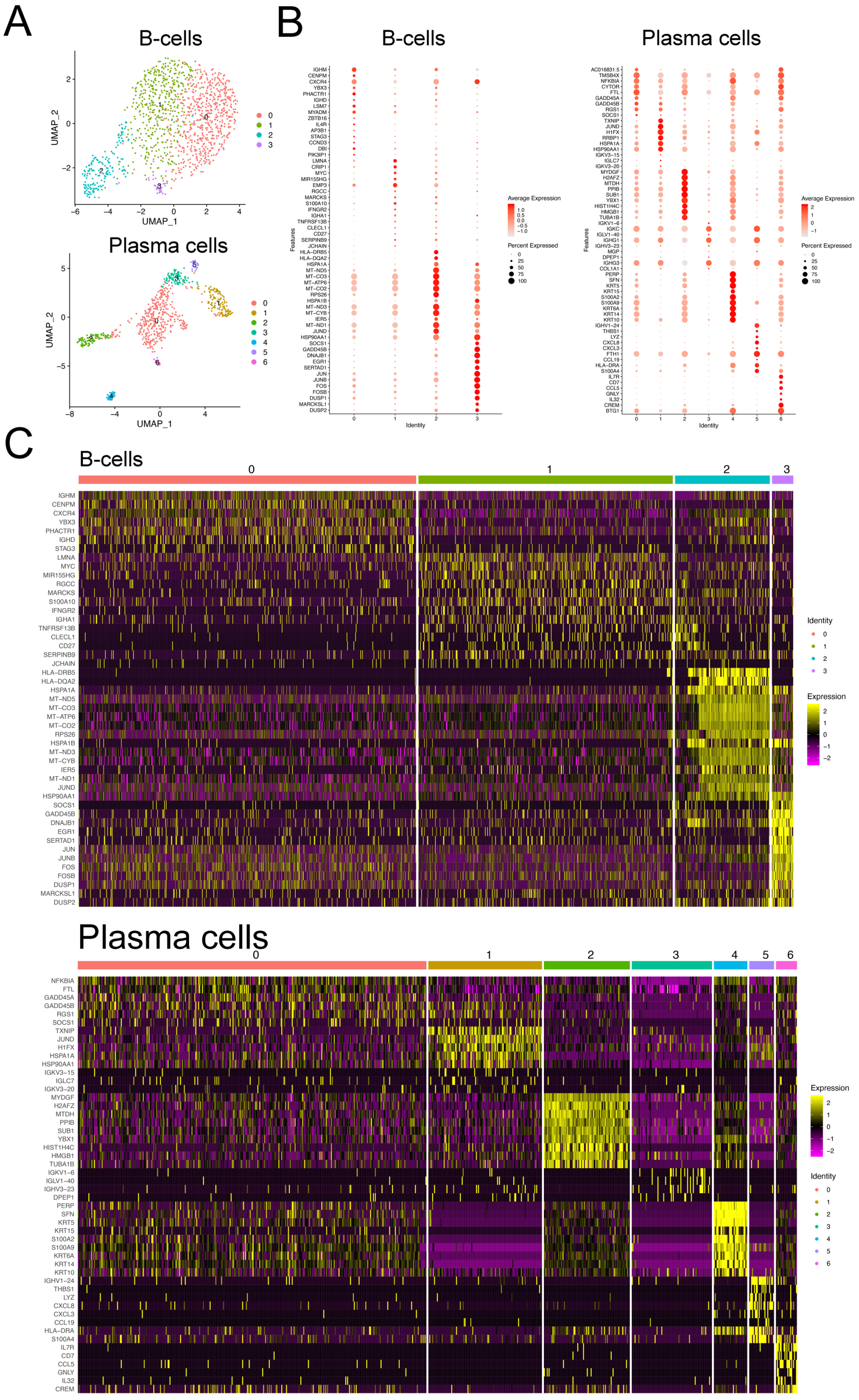

Supplemental Figure 6. B cell and Plasma cell subsets in HS skin.
Subclustering of T-cells in HS skin (A). Marker genes for each of the 4 B cell subcluster , and 6 Plasma cell subclusters (B). Heatmap of representative genes in each cluster (C).

Supplemental Table 1: Patient Demographics

	Sex	Age	BMI	Current or ex-smoker	Ethnicity	Age of HS onset	Hurley stage	Skin	Blood
HS patients									
**HS 01**	F	26	30.0	Yes	Caucasian	19	2	Yes	No
**HS 02**	F	40	33.9	Yes	Caucasian	15	3	Yes	Yes
**HS 03**	F	22	24.2	Yes	Caucasian	12	2	Yes	Yes
**HS 04**	F	46	40.5	Yes	Caucasian	27	2	Yes	Yes
**HS 05**	M	47	-	Yes	Caucasian	40	1	Yes	Yes
**HS 06**	M	40	38.5	Yes	Middle-Eastern	33	2	Yes	Yes
**HS 07**	M	53	21.9	Yes	Caucasian	45	3	Yes	Yes
**HS 08**	F	39	45.7	Yes	Middle-Eastern	20	2	Yes	Yes
**HS 09**	F	36	29.8	Yes	Caucasian	16	3	Yes	Yes
**HS 10**	F	15	36.2	No	Surinam-Creole	8	2	Yes	Yes
**HS 11**	M	41	28.6	Yes	Caucasian	10	2	Yes	Yes
**HS 12**	M	52	19.8	Yes	Caucasian	37	1	Yes	Yes
**HS 13**	M	46	39.0	Yes	Caucasian	16	3	Yes	Yes
**HS 14**	F	51	22.8	Yes	Surinam-Creole	20	2	Yes	Yes
**HS 15**	F	37	35.8	Yes	Middle-Eastern	30	2	Yes	Yes
**HS 16**	F	55	31.9	No	Caucasian	40	2	Yes	Yes
**HS 17**	M	56	41.4	No	Caucasian	12	2	Yes	Yes
**HS 18**	M	46	26.2	Yes	Middle-Eastern	40	2	Yes	Yes
**HS 19**	M	44	-	Yes	North-African	40	2	Yes	Yes
**HS 20**	M	31	25.7	Yes	Caucasian	22	2	Yes	Yes
**HS 21**	F	33	36.4	Yes	Surinam-Amerindian	20	2	Yes	Yes
**HS 22**	M	44	26.5	Yes	North-African	15	3	Yes	No
Controls									
**NN 01**	M	87	-	-	Caucasian			Yes	Yes
**NN 02**	M	73	-	-	Caucasian			Yes	Yes
**NN 03**	-	-	-	-	Caucasian			Yes	Yes
**NN 04**	M	66	23.4	-	Caucasian			Yes	Yes
**NN 05**	M	72	-	-	Caucasian			Yes	Yes
**NN 06**	F	78	-	-	Caucasian			Yes	Yes
**NN 07**	F	55	-	No	Caucasian			Yes	Yes
**NN 08**	F	74	-	-	Caucasian			Yes	Yes
**NN 09**	M	85	-	-	Caucasian			Yes	Yes
**NN 10**	F	62	20.9	Yes	Caucasian			Yes	Yes

Supplemental Table 2. DEGs in HS skin

Supplemental Table 3. DEGs in HS Blood

Supplemental Table 4: Cell-Cell Communications

Supplemental Table 5: Cytokine Response in Keratinocyte Clusters

Supplemental Table 6. BTK SYK Inhibitor treatment DEGs
